# The contribution of LARGE genomic rearrangements of BRCA1 and BRCA2 gene mutations in breast and ovarian cancer families in a clinical cohort

**DOI:** 10.1186/1897-4287-10-S2-A89

**Published:** 2012-04-12

**Authors:** S Sawyer, S Boyle, MA Young, S Kovalenko, R Doherty, J McKinley, K Alsop, M Rehfisch, S Macaskill, A Ha, V Beshay, G Lindeman, M Harris, S Fox, G Mitchell, P James

**Affiliations:** 1 Peter MacCallum Cancer Centre: Familial Cancer Centre, Australia; 2 Peter MacCallum Cancer Centre: Molecular Pathology Laboratory, Australia; 3 Royal Melbourne Hospital: Familial Cancer Centre, Australia; 4 Monash Medical Centre: Familial Cancer Centre, Australia

## Background

The use of multiplex ligation-dependent probe amplification (MLPA) to detect large scale rearrangements is now a standard component of *BRCA1* and *BRCA2* gene testing in the clinical setting. With the cost of full Sanger sequencing up to 4 times higher than the cost of MLPA, it is important not only to determine the prevalence of these mutations but to ascertain the probability that a family may harbour a large deletion or rearrangement in the *BRCA1* and *BRCA2* genes. Here we examine the incidence and clinical associations of genomic rearrangements in the *BRCA1* and *BRCA2* genes in a cohort of index cases from high risk breast and ovarian cancer families recruited from familial cancer centres (FCC).

## Method and results

The Victorian FCC Translational breast cancer cohort includes 1222 index cases identified from families who had been seen through one of the four FCCs in Victoria. Until 2007, standard BRCA tests did not include MLPA but instead used a variety of sequencing-based methods which included PTT, DHPLC and Sanger sequencing. Of these cases, 246 (20.1%) were found to carry a *BRCA1* or *BRCA2* mutation using sequencing-based methods. In a small proportion of cases MLPA was performed prior to study commencement based on clinical indications leading to the detection of 11 mutations. A total of 965 cases were found not to carry a *BRCA1* or *BRCA2* mutation through sequencing-based methods and were eligible for MLPA in the study. A hundred and nine cases were excluded from testing: 19 did not fit inclusion criteria, 93 had unsuitable DNA for testing.

In the remaining 856 cases a further 24 (2.8% of cases) *BRCA1* and *BRCA2* mutations were identified using MLPA. In the total cohort of 1113 index cases, 246 (22.1%) *BRCA1* and *BRCA2* mutations have been identified, including 36 (14.2% of mutations) large deletions and duplications detected through MLPA. Mean age of onset for first breast cancer diagnosis was 41 years (26-73) in mutation carriers detected using sequencing based methods and 40 years (18-60) in cases with genomic rearrangements detected by MLPA.

Analysis of the BRCAPRO scores revealed that the mean BRCA carrier probabilities for BRCA mutation carriers detected though MLPA were significantly higher than those detected using sequencing based methods (0.58 versus 0.37 respectively, p=0.002). Further analysis of correlations between mutation type and patient demographics including the cancer profile of the index case and their 1^st^ - 3^rd^ degree family members, rates of bilateral breast cancer, male breast cancer and early age of onset will be presented.

## Conclusion

MLPA detected genomic rearrangements accounted for 14% of all BRCA mutations in a large cohort of Victorian FCC families. The association with higher pre-test carrier probabilities indicates that an optimal strategy for BRCA mutation detection in which an initial MLPA screen in high risk families may avoid the need for sequencing in some patients where a genomic rearrangement is present, with an associated cost saving.

